# Development of PD3 and PD3-B for PDEδ inhibition to modulate KRAS activity

**DOI:** 10.1080/14756366.2022.2086865

**Published:** 2022-06-13

**Authors:** Jungeun Lee, Ho Jin Lee, Yeongcheol Lee, Bumhee Lim, Jongsik Gam, Dong-Chan Oh, Jeeyeon Lee

**Affiliations:** a College of Pharmacy and Research Institute of Pharmaceutical Sciences, Seoul National University, Seoul, Republic of Korea; b Department of Medicinal Bioscience, College of Interdisciplinary & Creative Studies, Konyang University, Nonsan, Republic of Korea; c Natural Products Research Institute, College of Pharmacy, Seoul National University, Seoul, Republic of Korea

**Keywords:** KRAS, PDEδ, fluorescent probes, affinity-based probe, KRAS relocalization

## Abstract

Despite extensive efforts over 40 years, few effective KRAS inhibitors have been developed to date, mainly due to the undruggable features of KRAS proteins. In addition to the direct approach to KRAS via covalent inhibition, modulation of the prenyl-binding protein PDEδ that binds with farnesylated KRAS has emerged as an alternative strategy to abrogate KRAS activity. For the verification of new therapeutic strategies, chemical probes with the dual functions of visualisation and pharmacological inhibition against oncogenic proteins are enormously valuable to understand cellular events related to cancer. Here, we report indolizino[3,2-c]quinoline (IQ)-based fluorescent probes (**PD3** and **PD3-B**) for PDEδ inhibition. By using the unique fluorescent characteristics of the IQ scaffold, a fluorescence polarisation (FP)-based binding assay identified **PD3** as the most effective PDEδ probe among the tested PD analogues, with a low *K*_d_ value of 0.491 µM and long retention time in the binding site of PDEδ. In particular, a FP-based competition assay using deltarasin verified that **PD3** occupies the farnesylation binding site of PDEδ, excluding the possibility that the FP signals resulted from non-specific hydrophobic interactions between the ligand and protein in the assay. We also designed and synthesised **PD3-B** (**5**), an affinity-based probe (ABP) from the **PD3** structure, which enabled us to pull down PDEδ from bacterial lysates containing a large number of intrinsic bacterial proteins. Finally, KRAS relocalization was verified in PANC-1 cells by treatment with **PD3**, suggesting its potential as an effective probe to target PDEδ.

## 1. Introduction

*RAS* is a well-known proto-oncogene and the most frequently mutated gene in various cancer types, such as pancreatic, colorectal, and lung cancer^1^. The mutations usually occur in codons G12, G13 or Q61, and most of them are missense gain-of-function mutations^2^. RAS protein is a GTPase that cycles between an active state (GTP-bound form) and an inactive state (GDP-bound form). The most well-known downstream pathways are the MAPK (RAF/MEK/ERK) and PI3K (PI3K/AKT/mTOR) signalling cascades, and the increased flux through downstream signalling is a key property of oncogenic mutation of RAS^3^^,^^4^. Despite extensive efforts over 40 years, there is only 1 FDA-approved RAS inhibitor, AMG-510 (sotorasib), which is mainly due to the undruggable feature of RAS proteins, including (1) high affinity for GDP and GTP and (2) lack of a well-defined hydrophobic pocket^5^^,^^6^. Recent advances provide directions for targeting RAS with direct approaches. The covalent inhibitor of KRAS G12C (AMG-510) was approved in May 2021 for the treatment of advanced or metastatic cancers and became the first approved drug directly targeting RAS. Additionally, another KRAS G12C inhibitor, MRTX-849, is in clinical trials^7^^,^^8^. However, other KRAS mutants have not been directly targeted by covalent inhibitors and elicited several groups to find alternative routes by inhibiting the protein that binds to KRAS^9^. Furthermore, inhibitors of farnesyltransferase (FT) showed therapeutic potential in a preclinical study, but their low efficacy in clinical studies hampered their further development as anticancer drugs^10^.

One alternative route to target KRAS is the inhibition of protein–protein interactions of KRAS with other proteins. Phosphodiesterase 6 delta subunit (PDEδ) is a prenyl-binding protein that is highly conserved in various species^11^. PDEδ was first found to be a noncatalytic subunit of PDE. Later, it was reported to interact with various proteins, including retinis pigmentosa GTPase regulator (RPGR)^12^, a large number of prenylated G proteins, such as Rac, Rap, Rhe, RAS and Rho^13^^,^^14^, and nonprenylated G proteins, such as Arl2 and Arl3^15^^,^^16^. PDEδ was identified as a trafficking chaperone of RAS subfamily proteins such as HRAS, NRAS, KRAS4a and KRAS4b and has been implicated in the regulation of the activity of prenylated RAS and other prenylated proteins by modulating their spatial localisation in cells^13^^,^^14^^,^^17^^,^^18^.

The enrichment of RAS on the plasma membrane (PM) is essential for signalling activity. This PM localisation is dependent on posttranslational modification (PTM) at the C-terminal hypervariable region (HVR) of RAS, which is required for binding to the PM^19^^,^^20^. All RAS proteins undergo farnesylation and carboxymethylation at HVR. The guanine nucleotide dissociation inhibitor (GDI)-like pocket of PDEδ directly binds to farnesylated RAS and stabilises the modified proteins in the cytosol. This process is essential for the PM localisation of RAS and RAS-mediated signalling to affect abnormal oncogenic signalling. In addition, the increased activity of PDEδ promotes RAS signalling by localising RAS at the plasma membrane^19^^,^^20^.

Thus, an efficient way to abrogate KRAS signalling is to block the distribution of KRAS by disrupting the interaction between farnesylated KRAS and PDEδ in the PDEδ binding site. Several small molecule inhibitors targeting PDEδ have been reported^21–28^. Deltarasin, the first reported PDEδ inhibitor, attenuated RAS oncogenic signalling by occupying the prenyl binding pocket of PDEδ and inhibited the proliferation of human pancreatic carcinoma cells both *in vitro* and *in vivo*^21^. In addition, the elevated expression of PDEδ has been reported in several human cancer cell lines, including breast, colon, and hepatocellular cancer cell lines^29–31^, and the level of PDEδ is strongly correlated with the expression of RAS as well as RAS activity in colorectal cell lines^30^. Taken together, these observations indicate that it is tremendously important to study the expression level, precise function, and cellular localisation of PDEδ by using chemical probes to verify the novel therapeutic strategy for KRAS.

Here, we report indolizino[3,2-*c*]quinoline (IQ)-based fluorescent probes (PDs) as a novel scaffold for PDEδ inhibition with low *K*_d_ values and long half-lives. By using the unique fluorescent characteristics of PDs, we confirmed the target specificity against PDEδ in bacterial lysate. We also investigated KRAS relocalization in PANC-1 cells by treatment with the **PD3**. Finally, **PD3-B**, a biotin-conjugated analog of **PD3**, was synthesised, which verified target engagement of **PD3** by pull down experiments.

## 2. Results and discussion

### 2.1. In silico docking study

**Figure 1.**
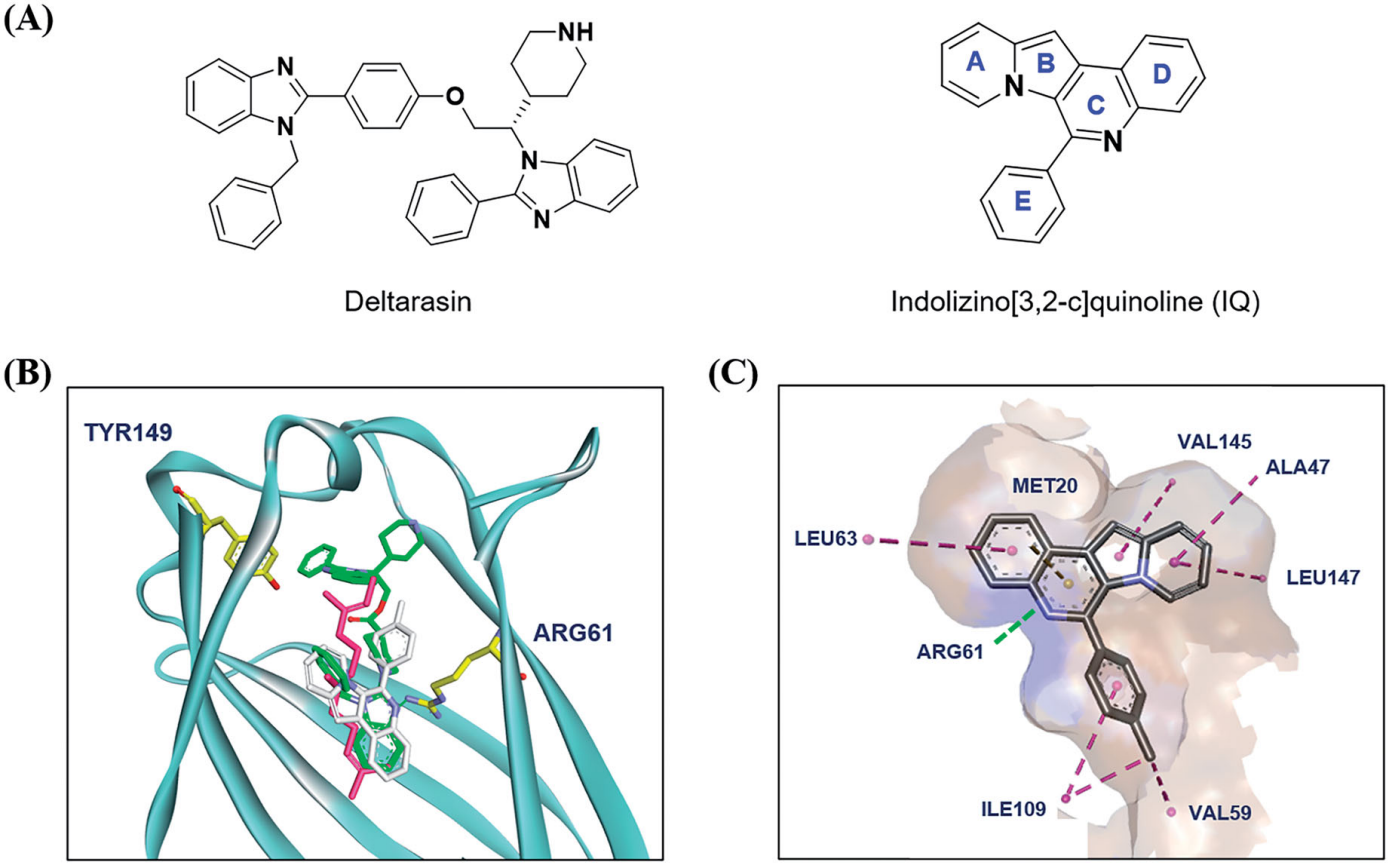
Design strategy of PDEδ probes by molecular docking study. (A) The chemical structures of Deltarasin and indolizino[3,2-c]quinoline (IQ) scaffold; (B) Superimposition of farnesyl (magenta), Deltarasin (green) and **PD3** (white) in the prenyl binding site of PDEδ. Farnesyl: obtained from a cocrystal complex with PDEδ (PDB: 3T5G), deltarasin: obtained from a cocrystal complex with PDEδ (PDB: 4JV8), **PD3**: obtained from a docking result in this study; (C) Predicted binding mode of **PD3** in the prenyl binding site of PDEδ. The surface of the PDEδ binding site was generated by hydrophobicity. Colour spectrum: the hydrophobic regions in brown and the hydrophilic ones in blue.

Our research group has developed a new chemical scaffold, indolizino[3,2-*c*] quinoline (IQ) derivatives, to explore their functions and biological applications^32–36^. During our initial in silico studies to identify the target proteins of IQs, we found that some IQ derivatives, named as PDs, could act as PDEδ probes due to their structural similarity to deltarasin (Figure 1(A)). To verify the possibility of PDs as probes of PDEδ, a molecular docking study was conducted on the prenyl binding site of PDEδ. The representative compound **PD3**, which has a basic chemical structure of an IQ scaffold, was docked to the crystal structure of PDEδ complexed with deltarasin (PDB: 4JV8). Then, the docked pose of **PD3** was overlaid with both the farnesyl group (PDB: 3T5G) and deltarasin (PDB: 4JV8) in the prenyl binding pocket of PDEδ (Figure 1(B)). **PD3** (white colour) was well overlaid with the deltarasin (green colour) as well as the farnesyl group (magenta colour) in the prenyl binding pocket of PDEδ. Interestingly, the hydrophobic part of the **PD3** was well matched with the hydrophobic region (shown in brown) of PDEδ (Figure 1(C)), and the hydrophilic part of **PD3** was also well matched with the hydrophilic region (shown in blue) of the PDEδ binding site. Binding of **PD3** is mediated by hydrophobic interactions with Met20, Ala47, Leu63, Val145, Leu147, Leu109 and Val59 and stabilised by hydrogen bonding between nitrogen on ring A and Arg61. These results indicated the possible use of PD derivatives as PDEδ probes.

### 2.2. Biochemical characterisations

#### 2.2.1. Fluorescence measurement

**Figure 2.**
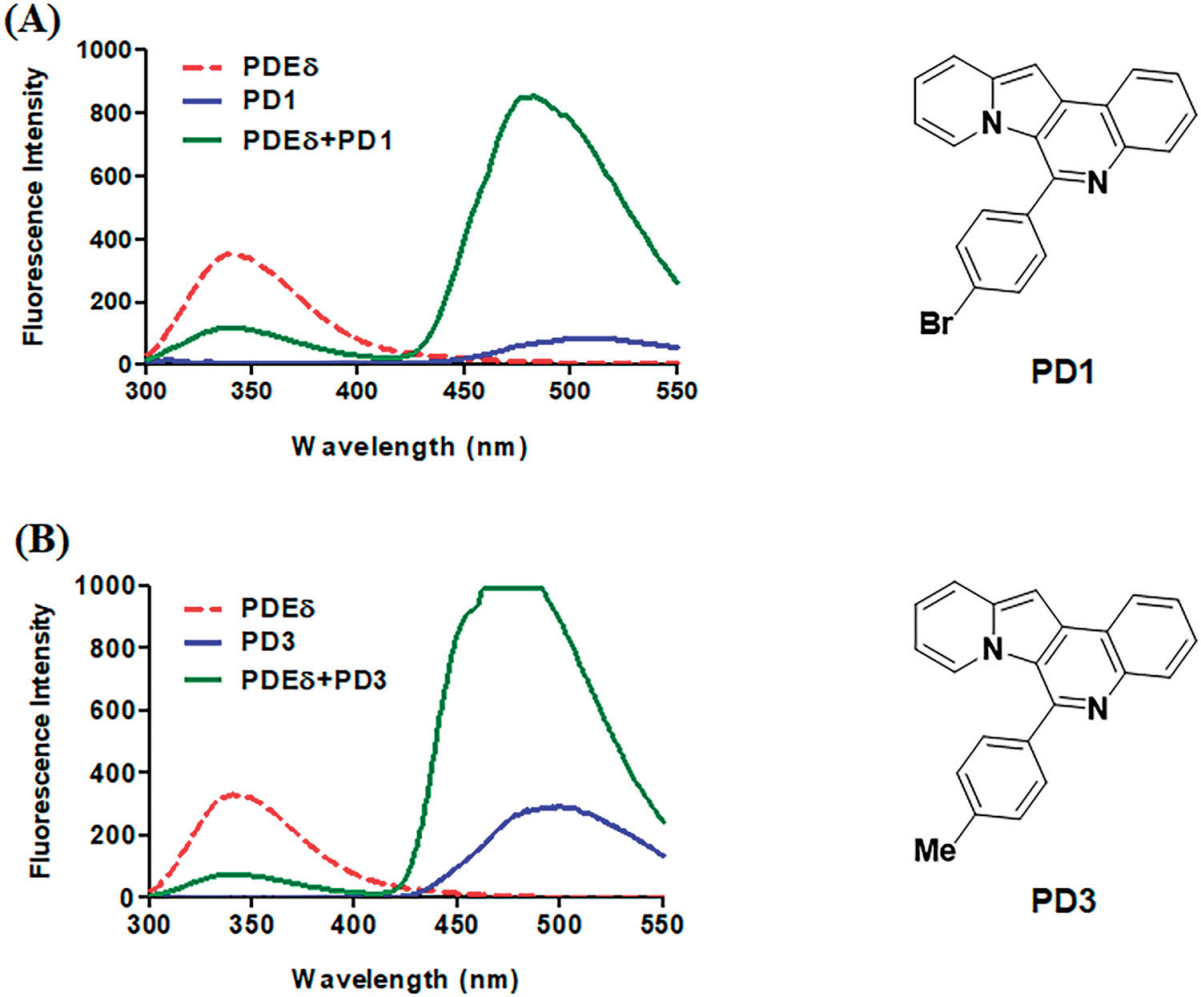
Representative emission spectra of **PD1** (A) and **PD3** (B). All emission spectra were taken in 20 mM Tris buffer (pH 7.5) at Ex 280 nm. Red dotted line: 2 μM PDE δ, blue line: 2 μM PD, green line: 2 μM PDE δ after incubation with 2 μM PD. Although the curve of **PD3** (B) was saturated, it was necessary to compare the binding abilities of each PD compound in the same condition as the initial screen.

With the structural rationale for the binding to PDEδ from the molecular docking studies, we measured the fluorescence responses of the PDs after incubation with recombinant PDEδ proteins to confirm the binding of PDs to PDEδ. We envisioned that the hydrophobic environment of the prenyl binding pocket of PDEδ, as depicted in Figure 1(C), could enhance the fluorescent signal due to the solvatochromic characteristics of IQ scaffold^32^. In addition, tryptophan residues near the binding site of PDEδ and the fluorophore of PDs could act as a fluorescence resonance energy transfer (FRET) pair. Hence, the fluorescence emission of PDs with or without PDEδ was measured upon excitation at 280 nm. All recoded spectra of PDs are depicted in Supplemental Figure S1. To compare the binding abilities of PD derivatives to the target protein, same concentration (2 µM) of PD compound was treated and then fluorescence intensity was measured in Spectrofluorometer FP-6500. Indeed, the fluorescence characteristics of PDs were significantly changed after incubation with PDEδ (Figure 2 and Supplemental Figure S1). The emission maxima of PDEδ at approximately 340 nm (red dotted line) decreased significantly after incubation with PDEδ, and the emission maxima of PDs at approximately 500 nm (blue line) in Tris buffer were shifted to 475 nm along with a drastic enhancement in fluorescence intensity (green line) upon excitation at 280 nm. Especially, some compounds (**PD3** and **PD6**) exceeded the maximum detection limit of Spectrofluorometer. These data suggested that a dramatic increase in the fluorescence intensity of PDs upon binding with PDEδ could be utilised to confirm the target specificity of PDs in bacterial lysates. Meanwhile, **PD2** and **PD10** were excluded from further study because of low fluorescence intensity (**PD2**) or lack of binding with PDEδ (**PD10**).

#### 2.2.2. *Determination of* K_d_

**Figure 3.**
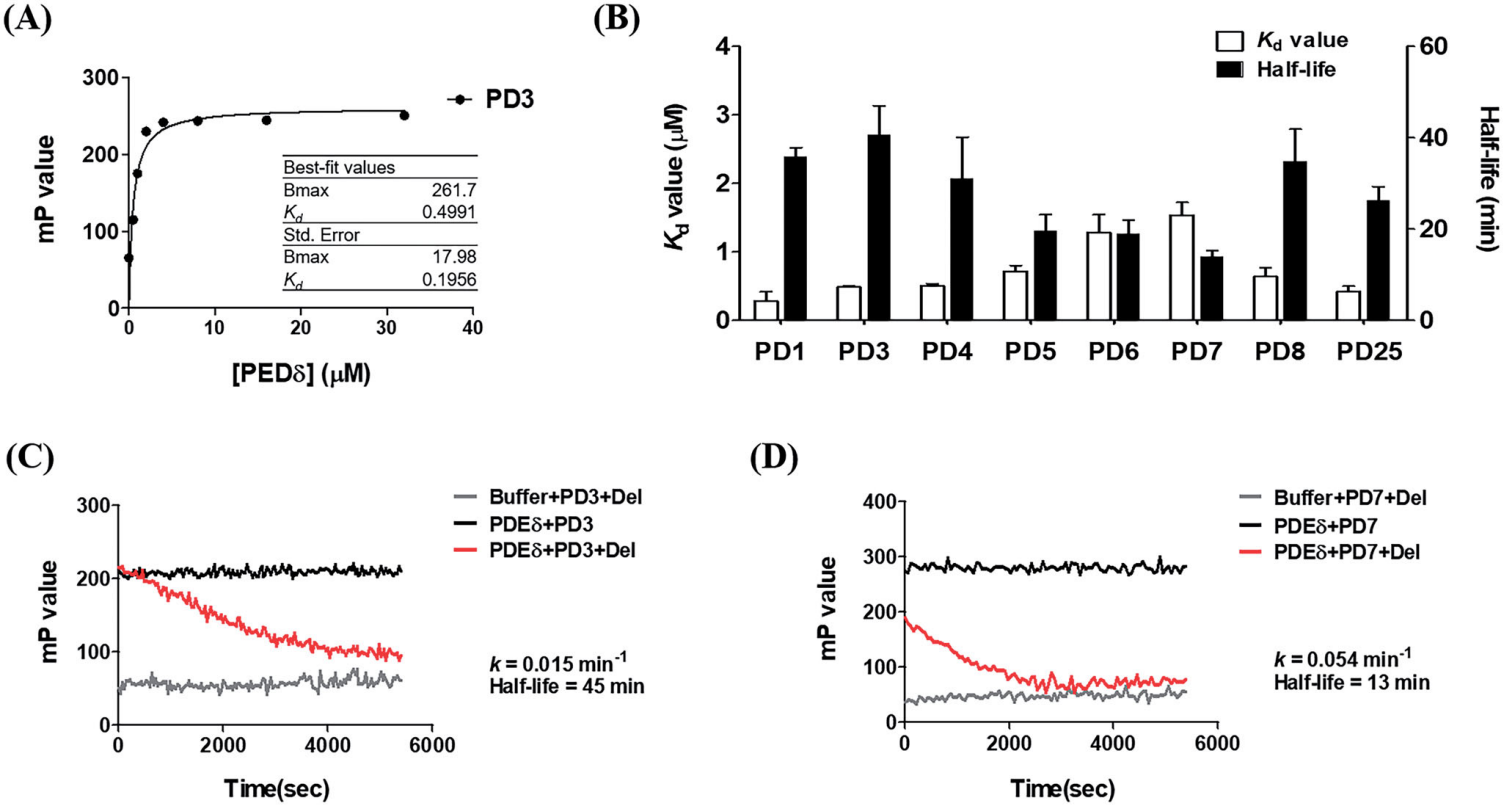
Binding affinities of PDs measured by fluorescence polarisation. (A) Representative binding curve of PDs (**PD3**) titrated with PDEδ. The FP value was measured in PBS buffer (pH 7.2) containing 8 concentrations (0, 0.5, 1, 2, 4, 8, 16 and 32 μM) of PDEδ mixed with 0.5 μM PDs. Ex: 440 nm, Em: 526 nm; (B) *K*_d_ value and half-life of each compound; (C, D) Kinetic trace of **PD3** and **PD7** in the competition assay. **PD3** was shown as a compound with the longest retention time (slow dissociation), whereas **PD7** was shown as a compound with the shortest retention time (fast dissociation). The half-life was obtained by a competition binding assay using excess deltarasin. The optimal concentration of PDEδ for the competition assay was determined using the *K*_d_ value and PD concentration (0.5 μM) for making more than 68% of the binding complex. Ex: 430 nm, Em: 520 nm.

To evaluate the binding affinities of PDs to PDEδ, we also carried out a fluorescence polarisation (FP)-based binding assay after incubation of PDs with PDEδ. Twenty-five compounds were screened based on their affinities to PDEδ, and *K*_d_ values were obtained in the range of 0.2–3.0 µM (Supplemental Figure S2). Figure 3(A) shows a representative hyperbolar binding curve of the PDs titrated with PDEδ. Fluorescence polarisation was efficiently induced with the unique optical properties of PDs as fluorescence probes. The absorption and emission maxima of the measured PD compounds are summarised in Table 1. In the presence of 0.5 µM PD compounds, the polarisation value (mP value) increased following treatment with purified PDEδ in a dose-dependent manner until treatment with excess PDEδ induced saturation of binding. Among the tested compounds, 8 compounds were selected for further evaluation and SAR analysis. The binding curves for the selected 8 compounds are shown with their *K*_d_ values in Supplemental Figure S3.

**Table 1.** Optical properties of PDs along with binding parameters to PDEδ. 
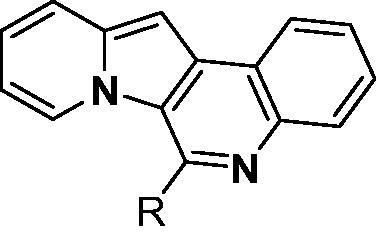

Compound	*R*	*λ_max ex_ (nm)	*λ_max em_ (nm)	**K_d_ value(μM)	**Half-life (min)	***AlogP
PD1	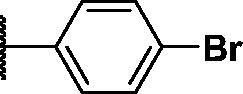	430	530	0.284 ± 0.140	36 ± 2	5.744
PD3	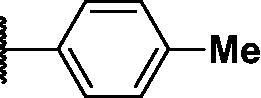	440	526	0.491 ± 0.012	41 ± 6	5.481
PD4	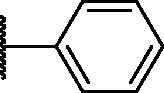	430	515	0.505 ± 0.033	31 ± 9	4.995
PD5	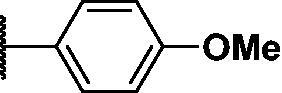	430	515	0.720 ± 0.085	20 ± 4	4.979
PD6	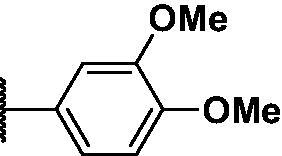	430	515	1.285 ± 0.261	19 ± 3	4.962
PD7	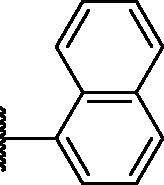	430	515	1.541 ± 0.180	14 ± 1	5.904
PD8	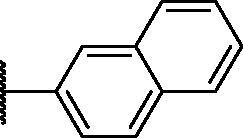	430	530	0.643 ± 0.128	35 ± 7	5.904
PD25	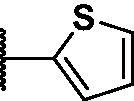	440	526	0.425 ± 0.075	26 ± 3	4.949

*These values represent maximum wavelengths of absorption and emission measured for each compound.

**Data are stated as the mean ± standard deviations (SD) of three independent experiments.

***AlogP was calculated using BIOVIA Discovery Studio 2020.

#### 2.2.3. Measurement of the half-life using competition assay

To confirm that the PD compound occupies the same farnesylation binding site as deltarasin in PDEδ and to exclude the possibility that the FP signal resulted from non-specific hydrophobic interactions, we carried out a FP-based competition assay. PDs (0.5 µM) were incubated with the purified PDEδ protein for 2 h to ensure complete binding between the compound and the protein. Then, deltarasin was added to replace the PD compound in the prenyl binding pocket of PDEδ, which decreased the FP due to the free rotation of the released PD compound. As shown in Figure 3(B), the reverse correlation between Kd values and half-lives was observed (Figure 3(B) and Table 1). In other words, compounds with tight binding exhibited longer retention times (half-lives) in the PDEδ protein when competing with deltarasin. The rate constants and half-lives were measured only when the increased concentration of deltarasin produced concentration-independent kinetic parameters, which reflects complete displacement of the binding site with the deltarasin. The kinetic trace was fit to a single exponential decay to generate the dissociation rate constant and half-life. Given that the *K*_d_ value is a ratio of *k*_off_/*k*_on_ in a simple bimolecular binding model, the measured half-life and *K*_d_ are not always matched due to the contribution from the association rate constant. Figure 3(C) (**PD3**) and 3 D (**PD7**) show a representative kinetic trace obtained in the competition assay. **PD3**, with a *K*_d_ value of 0.491 µM, showed much slower dissociation from the PDEδ protein (*k* = 0.015 min^−1^; half-life = 45 min) than **PD7**, with a *K*_d_ value of 1.541 µM (*k* = 0.054 min^−1^; half-life = 13 min).

#### 2.2.4. Structure-activity relationship analysis and docking studies of PDs

As shown in Table 1, bulky substituents at the R group showed decreased binding affinities with relatively high *K*_d_ values and short half-lives (**PD6** and **PD7**). **PD8** showed a higher affinity for PDEδ and a longer half-life than **PD7** (0.643 vs. 1.541 µM for *K*_d_; 35 vs. 14 min for half-life). As depicted in Figure 1, the binding pocket of PDEδ has a deep and narrow hydrophobic cleft, in which bulky groups cannot fit. Only –Br, –CH_3_ groups attached to the 4-position of the phenyl ring or smaller thiophen ring can fit in the binding site. The steric clash in the binding pocket impeded tight binding in the case of the naphthyl group attached at the 1 position (**PD7**), whereas **PD8** had a favourable interaction due to the different orientation of the naphthyl unit. The docking scores of **PD7** and **PD8** are well correlated with their binding affinities. **PD8**, which has higher binding affinity to PDEδ, has higher total scores along with lower clash compared to **PD7** (Supplemental Table S1). Additionally, PD analogues showed high lipophilicities with AlogP values between 4 and 6 (Table 1) and the calculated AlogP depends on the type of E ring of PDs. It is suggested that lipophilicity of PD compounds can be optimised by introducing various substituents at PD scaffold for further medicinal applications. Among the compounds, **PD3** showed the longest half-life and **PD1** showed the highest affinity for PDEδ. Between **PD3** and **PD1**, we selected **PD3** due to its higher emission intensity upon binding to the target and lower LogP value than those of **PD1**, suitable for investigating target specificity and engagement. Also, **PD1** has a bromo phenyl ring at the E ring, which may cause phototoxicity during cell-based assays.

### 2.3. Target validation of PD3 using in-gel fluorescence

**Figure 4.**
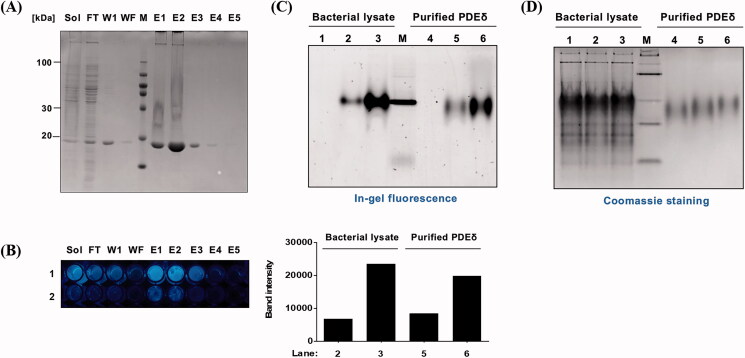
Evaluation of target specificity. (A) SDS–PAGE of fraction samples during the purification of PDEδ protein. Sol: Soluble, FT: Flow through, W1: Washing 1, WF: Washing final, M: Marker, E1–5: Elution 1-5, PDEδ: 20 kDa; (B) Fluorescence image of mixture of **PD3** and fraction samples on 96-well plate (Ex. 312 nm). Lane 1: 0.5 mM PD3. Lane 2: 0.1 mM **PD3**. (C) Top: fluorescence image of a 12% Native gel. Fluorescence was measured by ImageQuant^TM^ LAS 4000 with a 605DF40 EtBr filter (Ex. 312 nm). Lane 1: lysate with DMSO, Lane 2: lysate with 5 µM **PD3**, Lane 3: lysate with 50 µM **PD3**, M: marker, Lane 4: purified PDEδ with DMSO, Lane 5: purified PDEδ with 5 µM **PD3**, Lane 6: purified PDEδ with 50 µM **PD3**. Bottom: band intensity of fluorescence image. (D) Coomassie staining of the same 12% native gel.

Next, we assessed the target specificity of **PD3** using in-gel fluorescence. The fluorescence of **PD3** was examined after incubation with the fractions of protein samples that were obtained during the purification process for PDEδ protein from the bacterial lysate (Figure 4(A,B)). Among those fractions, eluates E1, E2, and E3 and the soluble lysate fraction showed high concentrations of PDEδ (20 kDa), whereas the washing final (wf) fraction did not contain PDEδ in SDS–PAGE (Figure 4(A)). Strong cyan fluorescence was detected in eluates E1, E2, and E3 and the soluble lysate fraction after treatment with 0.5 mM **PD3**, whereas the fluorescence of the washing 1 (w1) and wf fractions was hardly detected (Figure 4(B)).

We also conducted native gel electrophoresis to further verify the specific binding of **PD3** to native PDEδ using in-gel fluorescence scanning. Since the native gel does not use SDS, the tertiary structure of the protein would be retained during electrophoresis. We assumed that **PD3** bound within the tertiary structure would be identified on the native gel. **PD3** was incubated with bacterial lysate containing the overexpressed PDEδ protein (Figure 4(C,D), lanes 1–3) or purified PDEδ (Figure 4(C,D), lanes 4–6), and then these samples were run in a native gel to avoid denaturation of the protein. The in-gel fluorescence shown in Figure 4(C) (Ex: 312 nm, Em: 585–625 nm) was compared to the same gel stained with Coomassie that visualised the protein bands (Figure 4(D)), which confirmed the formation of the **PD3**-target protein complex. As expected, the fluorescence band was detected at the location of PDEδ and only detected in the lane with **PD3** (lanes 2, 3, 5, and 6). The quantification data of the in-gel fluorescence showed increased fluorescence intensity in a concentration-dependent manner in both PDEδ-overexpressing bacterial lysate (Figure 4(C) bottom, lanes 2 and 3) and purified PDEδ samples (Figure 4(C) bottom, lanes 5 and 6). Taken together, our gel fluorescence data revealed that **PD3** specifically binds to PDEδ in bacterial lysates that also contain a large number of other bacterial proteins.

### 2.4. Design and synthesis of PD3-B

To confirm whether PDs also specifically bind to PDEδ in the complex proteome of bacterial lysate, we designed **PD3-B** (**5**), an affinity-based probe (ABP) based on the **PD3** structure, for selective profiling of the target protein of **PD3**. **PD3**-B contains a biotin module that would furnish the strong biotin-streptavidin interaction for the pull-down experiment.

The preparation of biotin-conjugated **PD3-B** (**5**) is described in Scheme S1. The starting compound **PD3** (**1**) was synthesised as previously described^32^. First, iodine was introduced by NIS for the next coupling reaction. Then, the Pd-catalyzed Stille cross-coupling reaction between stannane and iodinated **PD3** (**2**) was conducted under reflux conditions. Next, the TMS group of **3** was deprotected by potassium carbonate in MeOH. Finally, biotin-PEG3-azide was conjugated with **4** by a copper(I)-catalyzed azide-alkyne cycloaddition (CuAAC) reaction using CuSO_4_ and sodium ascorbate to yield **PD3-B**.

### 2.5. Pull-down experiment

**Figure 5.**
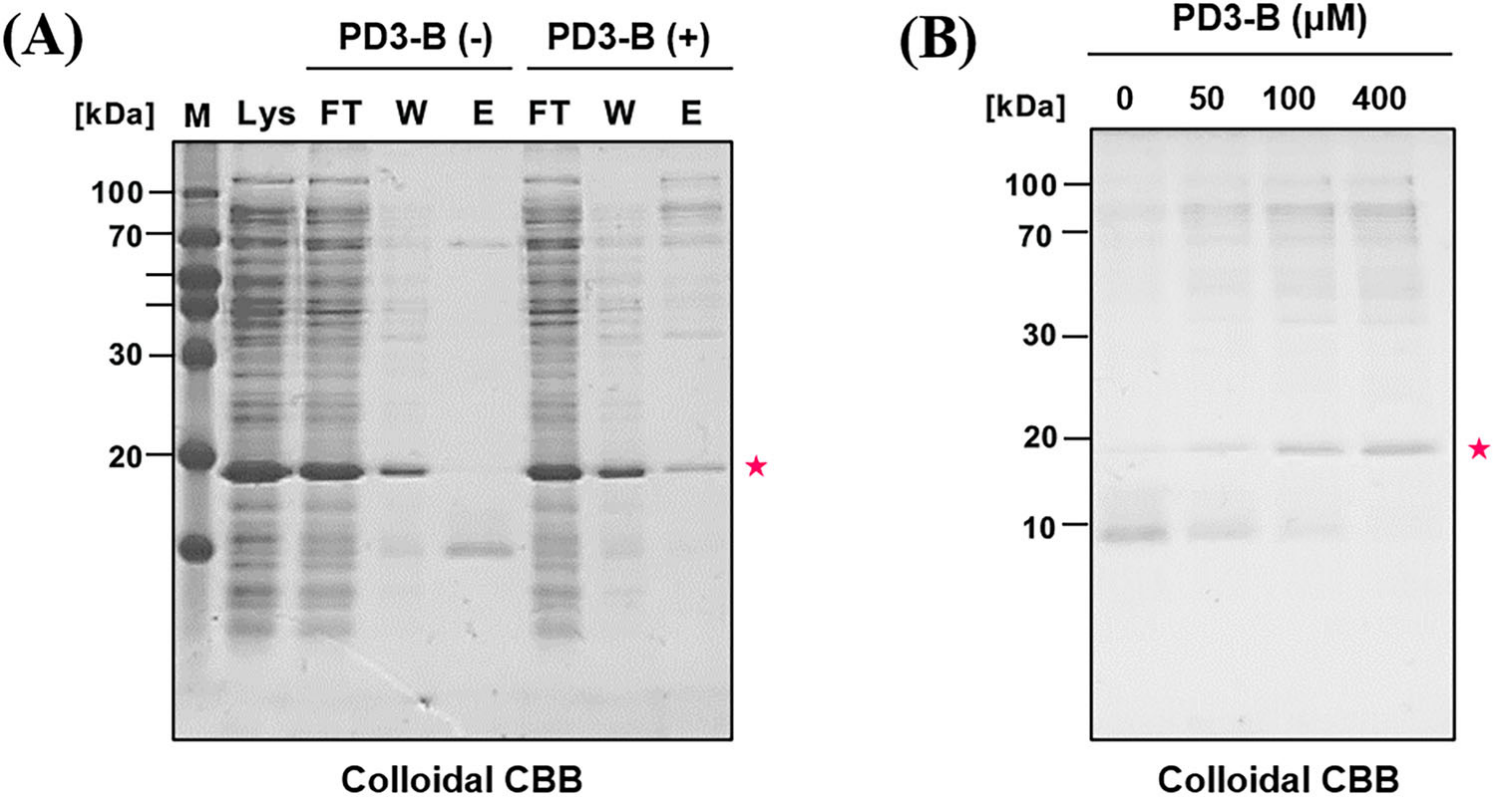
Affinity pull-down assay of bacterial lysate (PDEδ overexpressed) with **PD3-B**. NeutrAvidin resin was incubated with **PD3-B** (100 μM) followed by bacterial lysate (150 μg) in PBS buffer. Then, bound proteins were eluted by adding elution buffer (2% SDS in PBS) at room temperature. The eluted samples were subjected to SDS–PAGE (18% polyacrylamide gel). The gels were stained with colloidal Coomassie brilliant blue solution. (A) Colloidal CBB staining of SDS–PAGE gel. Lys: bacterial lysate, FT: flow through in step 1. W: washing sample in step 1, E1: elution sample in step 2. PDEδ: 20 kDa; (B) Concentration-dependent pull down of bacterial lysate by **PD3-B**.

We subsequently investigated whether **PD3-B** (**5**) specifically identifies PDEδ in the complex proteome of bacterial lysate using an affinity pull-down experiment (Figure 5). 100 µM **PD3-B** (**5**) was initially incubated with NeutrAvidin resin before incubation with bacterial lysate (150 µg) overexpressing PDEδ. For elution of the bound proteins, an elution buffer (2% SDS in PBS) was added to the resin and gently eluted at room temperature due to the noncovalent interaction between **PD3-B** (**5**) and the target protein PDEδ. The eluted samples were subjected to SDS–PAGE (18% polyacrylamide gel), and then the gels were stained with colloidal Coomassie brilliant blue solution to visualise the bound proteins. DMSO was used as a negative control. Surprisingly, PDEδ (20 kDa) was only detected in **PD3-B** (**5**) and not in the DMSO control lane after elution of the bound protein (Figure 5(A), lane E). In addition, the intensity of the PDEδ band increased depending on the concentration of **PD3-B** (**5**) (Figure 5(B)). The data suggested that **PD3** can bind to PDEδ with high selectivity for various proteins in the bacterial lysate.

Notably, the target protein band (PDEδ) of **PD3** was detected on the gel stained with colloidal Coomassie brilliant blue without the formation of a covalent bond. In general, an affinity-based probe (ABP) contains a functional group to generate a covalent bond with the target protein for tight binding. The binding affinity of **PD3-B** (**5**), a noncovalent ABP, was sufficiently tight to elicit the target protein in the pull-down experiments. These results are consistent with the observed high affinity (*K*_d_ = 0.491 µM) and long half-life (41 min) of **PD3** to PDEδ in the fluorescence polarisation assays.

### 2.6. Localisation change of KRAS by PD3

**Figure 6.**
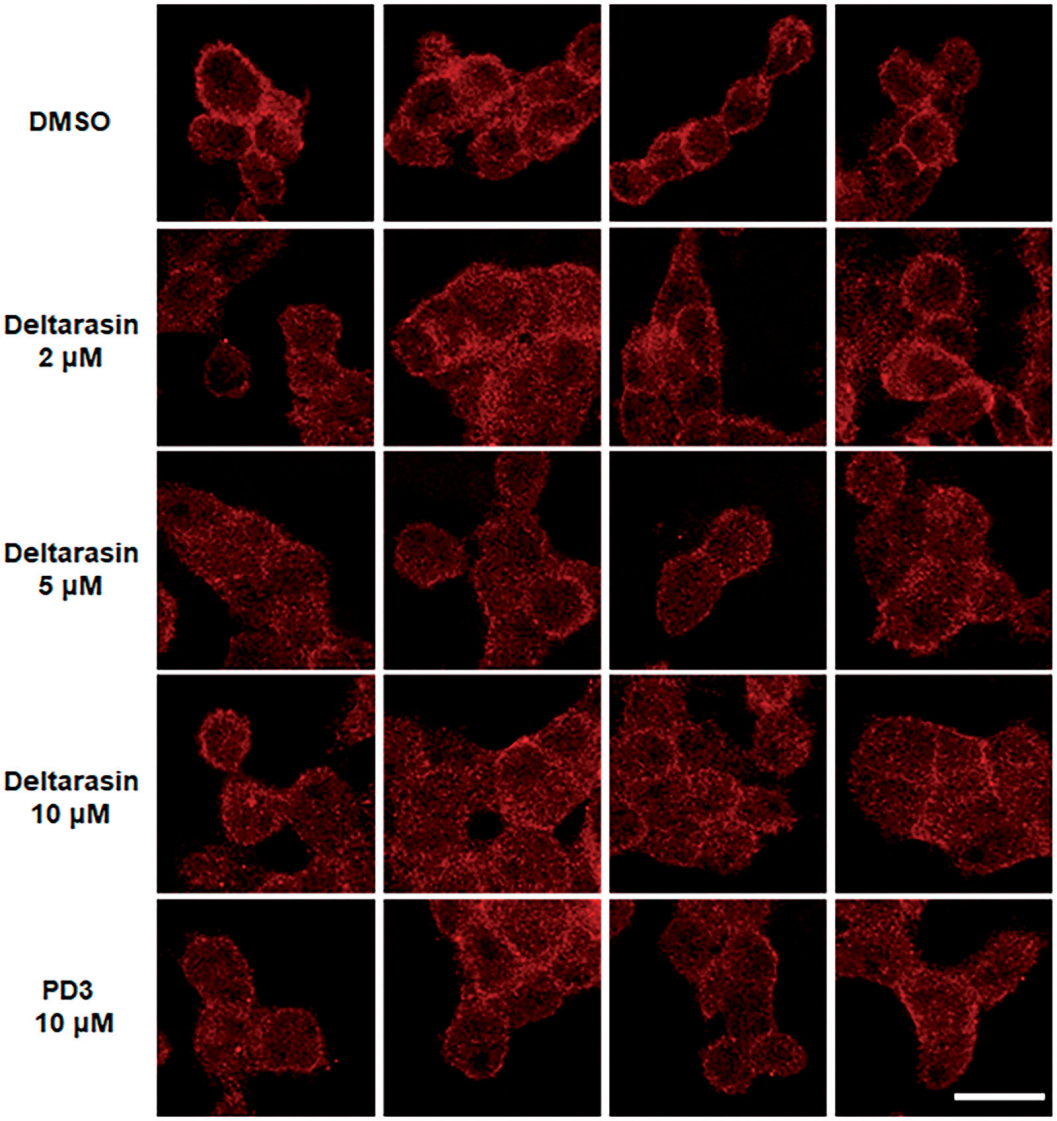
Immunofluorescence of PANC-1 cells with an anti-Pan-RAS antibody (red). DMSO was used as a negative control, and deltarasin was used as a positive control. Ex: 633 nm, Em: ≥638 nm. The scale bar represents 20 µm.

Finally, we investigated whether **PD3** changes KRAS localisation by inhibiting the interaction of KRAS with PDEδ in human pancreatic cancer cells. PDEδ binds the farnesylated RAS proteins and stabilises them^19^. Therefore, PDEδ activity leads to the accumulation of RAS at the plasma membrane (PM); conversely, downmodulation of PDEδ enhances RAS diffusion in the cytoplasm. To confirm the effect of **PD3** on the relocalization of RAS, we carried out immunofluorescence staining of PANC-1 cells with an anti-RAS antibody (Figure 6). As a positive control, treatment with deltarasin reduced the amount of RAS at the PM. Conversely, in the DMSO-treated control group, RAS was mainly localised in the PM of PANC-1 cells. In particular, the red fluorescence was effectively reduced at the PM by treatment with 10 µM **PD3** in the same way as the positive control deltarasin. It is supposed that **PD3** can induce changes in RAS localisation from the plasma membrane by interrupting the protein–protein interaction of KRAS-PDEδ.

## 3. Conclusion

In this work, we explored the PD series as novel chemical probes that target PDEδ to modulate KRAS activity. Our molecular docking studies suggested the possibility of PDs binding with PDEδ by demonstrating that the farnesyl group and deltarasin are well overlaid with PD in the prenyl binding site of PDEδ. Next, we checked the interaction of PDs with PDEδ by using the dramatic increase in PD fluorescence due to FRET. We also evaluated the binding affinity and kinetic parameters of PDs bound to PDEδ to quantify the interaction of the PD-PDEδ complex. When the *K*_d_ value was lowered, the half-life increased, and the SAR results were in accordance with the docking results.

Among the tested compounds, **PD3** showed a high affinity for PDEδ (*K*_d_ = 0.491 µM) with the longest half-life (41 min.). In addition, **PD3** specifically binds to PDEδ in the bacterial lysate during native gel electrophoresis. With sufficient binding affinity to PDEδ guaranteed, we designed **PD3-B** (**5**), an affinity-based probe from the **PD3** structure, for selective profiling of target proteins of **PD3**. We confirmed that **PD3-B** (**5**) can bind highly selectively to PDEδ out of various proteins in bacterial lysates in a pull-down experiment. Finally, our fluorescent imaging data revealed that **PD3** can induce changes in KRAS localisation from the plasma membrane by interrupting the protein–protein interaction of KRAS-PDEδ. Taken together, our results suggest that **PD3** and **PD3-B** (**5**) are effective chemical probes for PDEδ with high binding affinity and high selectivity.

## 4. Experimental

### 4.1. Chemistry

#### 4.1.1. General information for synthesis

Starting materials, reagents and solvents were purchased from Alfa Aesar (Ward Hill, MA), TCI chemicals (Tokyo, Japan) and Sigma–Aldrich (Saint Louis, MO). Both ^1^H and ^13 ^C NMR spectra were recorded on a JEOL JNM spectrometer (400 MHz for ^1^H NMR and 100 MHz for ^13 ^C NMR). Chemical shifts were expressed in ppm (δ) and were referenced to the residual solvent peak. Analytical thin-layer chromatography (TLC) was performed using precoated silica gel (E. Merck Kiesegel 60F254, layer thickness 0.25 mm), and chromatography was performed using silica gel 60 (40–60 µm). Mass spectra were recorded on a 6130 Single Quadrupole LC/MS (Agilent Technologies, Santa Clara, CA), and high-resolution mass spectra (HRMS) were collected under fast atom bombardment (FAB) conditions on a JMS-700 MStation (JEOL, Tokyo, Germany). HPLC analysis was performed on a YL9100 reversed-phase HPLC (Younglin, Gyeonggi-do, South Korea). The synthetic methods and optical properties of the IQ series were previously published by our research group^32^.

#### 4.1.2. Procedure for synthesis of 5 (PD3-B)

##### 4.1.2.1. 12-iodo-6-(p-tolyl)indolizino[3,2-c]quinoline (2)

A solution of the 6-(p-tolyl)indolizino[3,2-c]quinoline (130 mg, 0.42 mmol) in DCM (4 ml) was treated with N-iodosuccinimide (114 mg, 0.51 mmol). The mixture was stirred at room temperature for 2 h. The resulting residue was diluted with H_2_O and extracted with DCM followed by drying over Na_2_SO_4_. The solvent was removed and the residue was purified via column chromatography (SiO_2_, 10: 2: 1, hexane: DCM: EtOAc) to afford 12-iodo-6-(p-tolyl)indolizino[3,2-c]quinoline as a yellow solid (150 mg, 82%). ^1^H NMR (400 MHz, DMSO-d_6_) δ 9.56 (m, 1H), 8.12 (m, 1H), 7.85 (d, *J* = 7.2 Hz, 1H), 7.77 (m, 3H), 7.55 (d, *J* = 8.4 Hz, 2H), 7.46 (d, *J* = 8.4 Hz, 2H), 7.36 (m, 1H), 6.78 (m, 1H), 2.49 (s, 3H, overlapped with DMSO-d_6_ solvent peak); LCMS (ESI) m/z 435.0 [M + H]^+^.

##### 4.1.2.2. 6-(p-tolyl)-12-((trimethylsilyl)ethynyl)indolizino[3,2-c]quinoline (3)

A mixture of 12-iodo-6-(p-tolyl)indolizino[3,2-c]quinoline (70 mg, 0.16 mmol), tributyl(trimethyl-silylethynyl)tin (75 mg, 0.19 mmol), Pd(PPh_3_)_4_ (19 mg, 0.016 mmol) and CuI (3 mg, 0.016 mmol) was dissolved in 3.2 mL of anhydrous THF followed by stirring for 2 h at 80 °C. The solvent was removed by evaporation followed by purification with flash chromatography (SiO_2_, 10: 2: 1, hexane: DCM: EtOAc) gave 6-(p-tolyl)-12-((trimethylsilyl)ethynyl)indolizino[3,2-c]quinoline (50 mg, 77%) as a yellow oil. ^1^H NMR (400 MHz, DMSO-d_6_) δ 9.23 (dd, *J* = 8.0, 1.6 Hz, 1H), 8.12 (dd, *J* = 8.0, 1.6 Hz, 1H), 7.93 (d, *J* = 7.2 Hz, 1H), 7.84 (d, *J* = 8.8 Hz, 1H), 7.78 (td, *J* = 7.2, 1.6 Hz, 1H), 7.71 (td, *J* = 8.0, 1.2 Hz, 1H), 7.57 (d, *J* = 7.6 Hz, 2H), 7.44 (m, 3H), 6.86 (td, *J* = 6.8, 1.6 Hz, 1H), 2.49 (s, 3H, overlapped with DMSO-d_6_ solvent peak), 0.39 (s, 9H); LCMS (ESI) m/z 367.0 [M + H]^+^.

##### 4.1.2.3. 12-ethynyl-6-(p-tolyl)indolizino[3,2-c]quinoline (4)

To a solution of 6-(p-tolyl)-12-((trimethylsilyl)ethynyl)indolizino[3,2-c]quinoline (45 mg, 0.11 mmol) in 2 mL of MeOH was added K_2_CO_3_ (31 mg, 0.22 mmol), followed by stirring for 3 h at room temperature. The solvent was removed by evaporation and the resulting residue was diluted with H_2_O. The product was extracted with DCM, followed by drying over Na_2_SO_4_. The desired product was purified by column chromatography (SiO_2_, 10: 2: 1, hexane: DCM: EtOAc) to 12-ethynyl-6-(p-tolyl)indolizino[3,2-c]quinoline (20 mg, 54%) as a yellow solid. ^1^H NMR (400 MHz, DMSO-d_6_) δ 9.21 (dd, *J* = 8.0, 1.6 Hz, 1H), 8.11 (d, *J* = 8.0 Hz, 1H), 7.92 (d, *J* = 8.4 Hz, 1H), 7.88 (d, *J* = 8.4 Hz, 1H), 7.75 (m, 2H), 7.57 (d, *J* = 7.6 Hz, 2H), 7.47 (d, *J* = 7.6 Hz, 2H), 7.41 (m, 1H), 6.84 (m, 1H), 4.78 (s, 1H), 2.49 (s, 3H, overlapped with DMSO-d_6_ solvent peak); ^13 ^C NMR (100 MHz, DMSO-d_6_) δ 148.84, 143.61, 141.71, 139.36, 136.68, 130.19 (2 C), 129.91, 129.72, 129.06 (2 C), 128.67, 127.34, 126.92, 126.40, 123.58, 122.62, 120.27, 118.34, 112.70, 87.41, 87.18, 78.28, 21.62; LCMS (ESI) m/z 333.10 [M + H]^+^; HRMS (FAB) m/z calcd for C_24_H_17_N_2_ 333.1392 ([M + H]^+^), found 333.1398.

##### 4.1.2.4. 5-((3aS,4R,6aR)-2-oxohexahydro-1H-thieno[3,4-d]imidazol-4-yl)-N-(2–(2–(2-(2-(4–(6-(p-tolyl) indolizino[3,2-c]quinolin-12-yl)-1H-1,2,3-triazol-1-yl)ethoxy)ethoxy)ethoxy)ethyl)pentanamide (5)

To a solution of 12-ethynyl-6-(p-tolyl)indolizino[3,2-c]quinoline (10 mg, 0.03 mmol) in 0.5 mL of t-BuOH and 0.5 ml of H_2_O was added CuSO_4_·5H_2_O (4 mg, 0.02 mmol), sodium ascorbate (6 mg, 0.03 mmol) and Azide-PEG3-biotin (13 mg, 0.03 mmol). The resulting mixture was stirred for 5 h at room temperature. The solvent was removed by evaporation and the residue was purified via column chromatography (SiO_2_, 10: 1, DCM: MeOH). The final mixture purified by preparative HPLC (H_2_O with 0.1% TFA/ACN with 0.1% TFA, 80/20 to 0/100 in 55 min, flow rate = 1.0 ml/min) afforded pure 5-((3aS,4R,6aR)-2-oxohexahydro-1H-thieno[3,4-d]imidazol-4-yl)-N-(2–(2–(2-(2-(4–(6-(p-tolyl) indolizino[3,2-c]quinolin-12-yl)-1H-1,2,3-triazol-1-yl)ethoxy)ethoxy)ethoxy)ethyl)pentanamide (3.6 mg, 15%) as a yellow solid. ^1^H NMR (400 MHz, MeOH-d_4_) δ 8.53 (s, 1H), 8.43 (dd, *J* = 8.4, 0.8 Hz, 1H), 8.10 (m, 2H), 7.88 (m, 2H), 7.82 (d, *J* = 8.4 Hz, 2H), 7.69 (m, 4H), 7.05 (td, *J* = 6.8, 1.6 Hz, 1H), 4.85 (m, 2H, overlapped with water peak), 4.43 (m, 1H), 4.22 (m, 1H), 4.07 (t, *J* = 4.8 Hz, 2H), 3.73 (m, 2H), 3.64 (m, 2H), 3.53 (m, 2H), 3.42 (m, 2H), 3.30 (m, 6H, overlapped with MeOH-d_4_ solvent peak), 3.11 (m, 3H), 2.85 (m, 1H), 2.63 (m, 1H), 2.60 (s, 3H), 2.04 (t, *J* = 7.2 Hz, 2H), 1.55 (m, 4H), 1.31 (m, 2H); ^13 ^C NMR (100 MHz, MeOH-d_4_) δ 174.54, 164.69, 145.56, 144.24, 143.34, 138.02, 134.09, 131.70, 131.61, 130.91, 130.87 (2 C), 128.62 (2 C), 127.97, 127.52, 127.06, 126.44, 125.28, 120.89, 119.90, 119.63, 118.25, 114.71, 101.61, 70.22, 70.14, 70.06, 69.82, 69.11, 69.04, 61.97, 60.26, 55.60, 50.53, 39.68, 38.81, 35.23, 28.30, 28.08, 25.43, 20.39; LCMS (ESI) m/z 777.34 [M + H]^+^; HRMS (FAB) m/z calcd for C_42_H_49_N_8_O_5_S 776.3547([M + H]^+^), found 777.3559.

### 4.2. Computational study

Molecular modelling study was carried out with the Sybyl-X 2.1.1 (Tripos Inc, St Louis, MO). The X-ray structure of human PDEδ (PDB ID: 4JV8) complexed with rac-S1 was retrieved from the RCSB (Research Collaboratory for Structural Bioinformatics) Protein Data Bank and the protein structure was prepared for docking studies. All water molecules and crystallised ligands were removed and hydrogen atoms were added to the crystal structure. The energy minimisation of protein was conducted using gradient minimisation (Powell’s method) applied the Tripos force field when the RMSD reached 0.001 kcal/mol·Å. 2 D structure of the docking ligands were drawn by ChemBioDraw ultra 13.0 (CambridgeSoft Corporation, Cambridge, MA) and optimised using “Ligand Preparation” in Sybyl-X 2.1.1. Docking experiments were performed by Surflex-Dock GeomX mode. The protomol was generated with a threshold parameter of 0.5 Å and a bloat parameter of 0 Å. The docking result was validated by examination of the RMSD of the re-docked ligand (rac-S1/Deltarasin) compared to the co-crystallised ligand. Binding mode were further analysed using Discovery Studio 4.0 Visualiser (Dassault Systèmes, San Diego, CA).

### 4.3. Biochemical experiment

#### 4.3.1. Cloning

The cDNA encoding human PDEδ was purchased from the Korea Human Gene Bank (Daejeon, South Korea). The corresponding DNA oligomers contained a NheI or BamHI restriction site were synthesised by Cosmogenetech Inc. (Seoul, South Korea). A forward (5′-GGTTGCTAGCATGTCAGCCAAGGACGAGCG-3′) and a reverse (5′-GGTTGGATCCTCAAACATAGAAAAGTCTCACTCTGGATGTGC-3′) primer were used for the PCR amplification. The resulting PCR fragments were digested with NheI (NEB, Ipswich, MA) or BamHI (NEB, Ipswich, MA) and ligated together with a pET28a(+) vector cut that contained the same restriction enzyme cleavage using T4 ligase (NEB, Ipswich, MA). The sequence of resulting clone was verified and transformed into the E. coli BL21(DE3) strain.

#### 4.3.2. Protein expression and purification

The transformed BL21(DE3) cells were grown in a rotary shaker at 37 °C to a density of 0.8 (OD600), and the protein expression was induced with 0.2 mM of isopropyl β-D-thiogalactopyranoside (IPTG) at 18 °C for 16 h. The resulting cell pellet was resuspended in 25 mM tris(hydroxymethyl)aminomethane (Tris) buffer [pH 8.0, 500 mM NaCl, 10 mM imidazole, 10% Glycerol, 1:100 protease inhibitor cocktail (Roche, Penzberg, Upper Bavaria, Germany)] and cells were lysed by sonication. Cell debris were removed by centrifugation and PDEδ was purified from supernatants through Nickel-nitrilotriacetic (Ni+-NTA) acid affinity chromatography (Qiagen, Hilden, Düsseldorf, Germany). The protein was eluted in 25 mM Tris buffer (pH 8.0, 500 mM NaCl, 250 mM imidazole 10% glycerol). After elution, the proteins were subsequently dialysed into 25 mM Tris buffer (pH 7.5, 500 mM NaCl, 10% glycerol, 1 mm DTT) for 4 h and concentrated by centrifugation.

#### 4.3.3. Fluorescence measurement

The fluorescence changes were measured to determine the interaction between PD compounds and PDEδ protein. The fluorescence spectra were obtained under Tris buffer [20 mM Tris-HCl (pH 7.5), 100 mM NaCl] at 20 °C. The concentration of PDEδ was 2 µM. After the addition of 2 µM PD compound, the mixture was gently inverted 3 times and then incubated for 1 min. The emission spectra were recorded on Spectrofluorometer FP-6500 (JASCO, Tokyo, Japan) at Ex 280 nm. The band width was 3 nm for excitation and 5 nm for emission.

#### 4.3.4. Fluorescence polarisation assay

PDEδ was diluted to make a series of two-fold dilutions with a starting concentration of 32 µM. The diluted solution was mixed with PBS buffer [137 mM NaCl, 2.7 mM KCl, 10 mM Na_2_HPO_4_, 2 mM KH_2_PO_4_ (pH 7.2)] and then the mixture was loaded on a 96 well black plate (SPL life Science, Gyeonggi-do, Republic of Korea). Compounds were transferred to the wells of assay plate and the final concentration of compounds was fixed to 0.5 µM. After the addition of the compound, the assay plates were incubated for 2 h at 4 °C. FP values were detected by SpectraMax M5 microplate reader (Molecular Devices, San Jose, CA) at the maximum absorption and emission wavelengths of PD compounds in assay buffer. *K*_d_ values were determined by previously reported method^37^.

#### 4.3.5. Competition binding assay

Deltarasin, a well-known PDEδ inhibitor, was purchased from Chemietek (Indianapolis, IN) for the competition assay. Assay buffer and the plate were the same as used in fluorescence polarisation assay. The optimal concentration of PDEδ was determined based on the *K*_d_ values and ligand concentration (0.5 µM) to ensure the formation of ligand-protein complex. The mixture of PDEδ and compounds were incubated for 2 h at 4 °C. After adding deltarasin to the mixture, FP was immediately recorded by SpectraMax M5 microplate reader (Molecular Devices, San Jose, CA) with excitation at 430 nm and emission at 520 nm. In SpectraMax M5 microplate reader, single excitation and emission wavelengths need to be used for testing all compounds. Therefore, Ex 430/Em 520 was used for competition binding assay. The selected ex/em wavelengths have only a difference of less than 10 nm from the maximum wavelengths, and there was no critical issue for conducting assay. The kinetic trace was fit to a single exponential decay to generate dissociation rate constant and half-life.

#### 4.3.6. Target specificity

The binding specificity of the **PD3** was measured using the protein fractions that can be obtained during purification of PDEδ. 0.5 mM **PD3** was mixed with each fraction: soluble, flow through, washing, washing final and elution 1–5 fractions. The mixture was transferred to the 96 well clear bottom plate (SPL life Science, Gyeonggido, Republic of Korea). UV light (312 nm) was irradiated by transilluminator (Vilber, Lourmat, France) and then the image was detected by Cannon EOS 550 D camera. The mixture of **PD3** and each fraction was separated by 21% SDS-PAGE separated, followed by in-gel fluorescence measurement using ImageQuant^TM^ LAS 4000 (GE Healthcare Life Science, Illinois, CA) with a 605DF40 EtBr filter. The equipment has a limited number of filters for fluorescence measurement, so we selected the most suitable filter for the measurement.

#### 4.3.7. Native gel analysis

**PD3** (5 or 50 µM) was added to the bacterial lysate (390 µM) or purified PDEδ protein (22.9 µM) and the mixture was incubated for 2 h at 4 °C. The samples were loaded on each lane and separated by 12% native PAGE in a running buffer [25 mM Tris-HCl (pH 8.0), 500 mM NaCl, 10 mM imidazole, 10% Glycerol] with 1/4 pellet of protease inhibitor (Roche, Penzberg, Upper Bavaria, Germany). The gels were run at 70 V for 2.5 h, followed by in-gel fluorescence measurement. The same gel was stained by Coomassie brilliant blue to visualise protein bands.

#### 4.3.8. Pull down experiment

NeutrAvidin UltraLink resin (100 µL, Thermo, Waltam, MA) was washed with PBS buffer (300 µL) 3 times before use. **PD3-B** (100 µM) in PBS buffer (500 µL) and NeutrAvidin UltraLink resin were incubated for 2 h at rt in Pierce Spin Columns (Thermo Fisher Scientific, Waltam, MA) with gentle shaking (40 rpm) and then washed with washing buffer (0.05% SDS in PBS buffer, 300 µL and 4 times). The beads were coated with BSA solution (500 µL) for 1 h at 4 °C with 40 rpm shaking, followed by washing with washing solution. The beads were incubated with bacterial lysate overexpressing PDEδ (150 µg) for overnight at 4 °C with 40 rpm shaking. After completed incubation, samples were centrifuged at 2000 rpm for 2 min, the flow through sample was removed, and the beads were washed with following conditions: 2 times with 0.1% SDS in PBS (300 µL), once with 2 M Urea in PBS (300 µL) and three times with PBS (300 µL). Bound proteins were eluted in an elution buffer (2% SDS in PBS) for 5 min. at room temperature followed by centrifugation (2000 rpm, 2 min). The samples were heated to 95 °C for 5 min in 1X standard SDS loading buffer and loaded on a 18% polyacrylamide gel and run at 150 V. The gels were stained by colloidal Coomassie brilliant blue solution for overnight at room temperature. DMSO was used for negative control for pull down experiment.

#### 4.3.9. Immunofluorescence staining

PANC-1 cells were seeded with cover slip in a 6-well cell culture plate (SPL life Science, Gyeonggi-do, Republic of Korea) and incubated for 24 h under 37 °C and 5% CO_2_ prior to the experiment. Subsequently, the negative control DMSO, positive control deltrasin (2, 5, 10 µM) and test compound **PD3** (10 µM) were processed for 18 h. Cells were washed 3 times with PBS [137 mM NaCl, 2.7 mM KCl, 10 mM Na_2_HPO_4_, 2 mM KH_2_PO_4_ (pH 7.2)] and fixed with 4% paraformaldehyde for 5 min. Then, cells were washed additional 3 times with PBS and permeabilized with PBS/0,1% Triton for 5 min. After an additional PBS washing step, cells were incubated in blocking buffer (5% BSA, 0.3% Triton X-100/PBS) for 1.5 h and washed 3 times with PBS before incubation with anti-pan RAS mouse monoclonal antibody (Calbiochem, San Diego, CA; OP40-100UG; 1:200) in blocking buffer. After 2 h incubation, cells were washed 3 times with PBS/0.1%Tween-20 and incubated with an Alexa-647 Donkey-anti-Mouse antibody (Invitrogen, Waltham, MA; 1:1000) as a secondary antibody. All steps were performed at room temperature. After final washing step with PBS/0.1%Tween-20, cells were stored in PBS for microscope. The fluorescence images were detected by confocal microscope (TCS-SP8 confocal laser scanning microscope, Leica, Germany). Fluorescence signal was obtained by excitation at 633 nm and emission at ≥638 nm.

## Supplementary Material

Supplemental Material
