# Supplementary material for: Development of PD3 and PD3-B for PDEδ inhibition to modulate KRAS activity
Source: J Enzyme Inhib Med Chem. 2022 Jun 13;37(1):1656–66. doi: 10.1080/14756366.2022.2086865 (PMC9225715; doi:10.1080/14756366.2022.2086865)

Supplementary data

Development of PD3 and PD3-B for PDEδ inhibition to modulate KRAS activity

Jungeun Lee[a], Ho Jin Lee[a], Yeongcheol Lee[a], Bumhee Lim[a], Jongsik Gam[b], Dong-Chan Oh[c], Jeeyeon Lee[a]*

[a]College of Pharmacy and Research Institute of Pharmaceutical Sciences, Seoul National University, Seoul, Republic of Korea; [b]Department of Medicinal Bioscience, College of Interdisciplinary & Creative Studies, Konyang University, Nonsan, Chungnam, Republic of Korea; [c]Natural Products Research Institute, College of Pharmacy, Seoul National University, Seoul, Republic of Korea

*Corresponding author. E-mail address: jyleeut@snu.ac.kr (J. Lee).

Contents:

1. Figure S1--S3

2. Figure S2--S4

3. Figure S3--S5

4. Table S1---S6

5. Scheme S1--S7

6. ^{1}H NMR, ^{13}C NMR spectra for **4**---S8

7. ^{1}H NMR, ^{13}C NMR spectra for **5** (**PD3-B**)--- S9

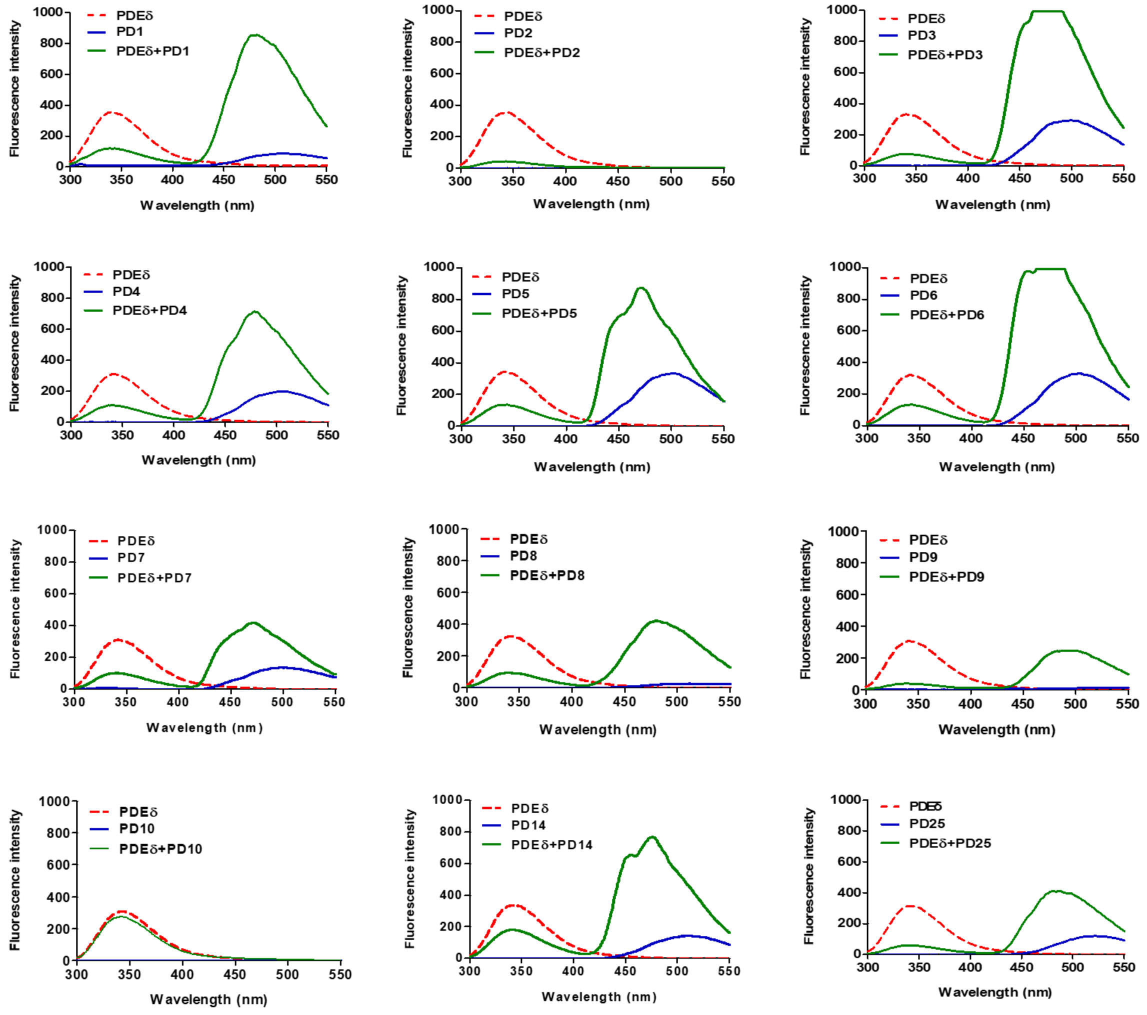

Fig. S1. Fluorescence changes in PD compounds after incubation with PDEδ. All emission spectra were taken in 20 mM Tris buffer (pH 7.5) at Ex 280 nm. Red dotted line: 2 μM PDE δ, blue line: 2 μM PD compound, green line: 2 μM PDE δ after incubation with 2 μM PD.

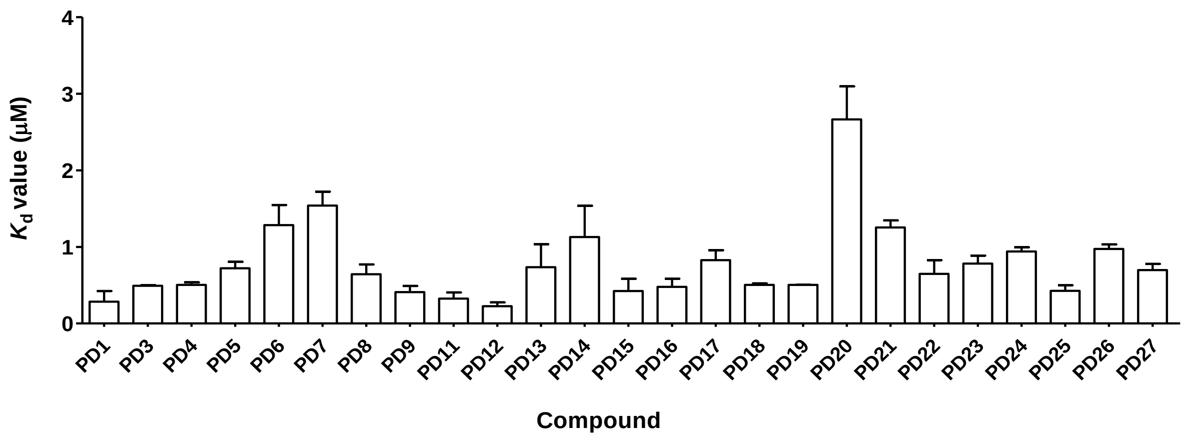

Fig. S2. K_d values of PDs. The FP value was measured in PBS buffer (pH 7.2) containing 8 concentrations (0, 0.5, 1, 2, 4, 8, 16 and 32 μM) of PDEδ mixed with 0.5 μM PDs. Ex: 440 nm, Em: 526 nm.

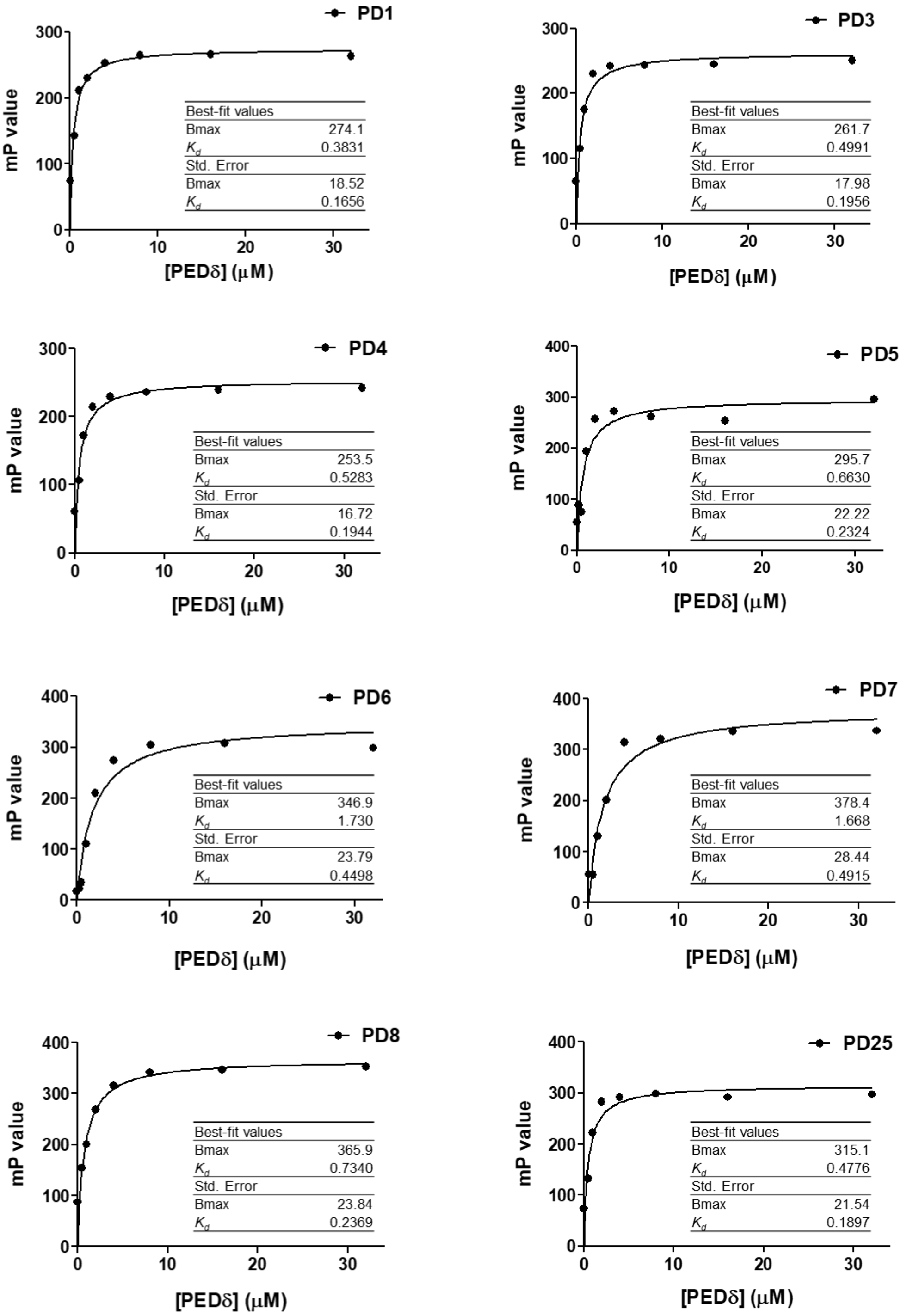

Fig. S3. The binding curves for the selected 8 compounds. FP values were collected in PBS buffer (pH 7.2) containing 8 concentration points (0, 0.5, 1, 2, 4, 8, 16 and 32 μM) of PDEδ mixed with 0.5 μM PDs. The values were measured at the maximum absorption and emission wavelengths of PD compounds in assay buffer, as shown in Table 1.

Table. S1. Docking scores of PDs (PDB: 4JV8).

Compound	Total score	Clash	Polar
Original ligand	8.0836	-0.6853	1.1295
**PD1**	5.8887	-3.0565	1.1674
**PD3**	6.9050	-2.9751	1.1564
**PD4**	6.2327	-2.2305	1.1713
**PD5**	6.5112	-3.653	1.1423
**PD6**	6.2321	-5.3844	1.021
**PD7**	5.5653	-5.0951	1.1783
**PD8**	6.6916	-4.6248	1.1779
**PD25**	6.3098	-1.5335	1.1713

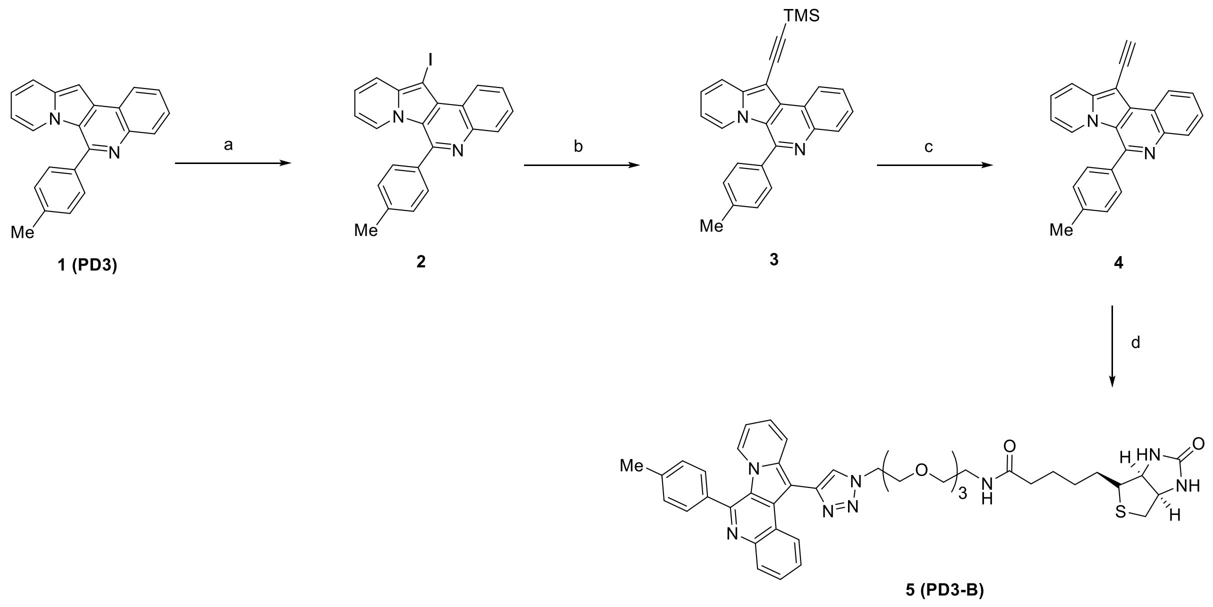

Scheme S1. Reagents and conditions: a) NIS, DCM, rt, 2 h; b) $Pd(PPh_3)_4$, CuI, tributyl (trimethylsilylethynyl)tin, THF, 80 °C, 2 h; c) K_2CO_3, MeOH, rt, 3 h; d) biotin-PEG_3-azide, $CuSO_4 \cdot 5H_2O$, sodium ascorbate, t-BuOH, H_2O, rt, on.

^{1}H NMR, ^{13}C NMR spectra for **4**

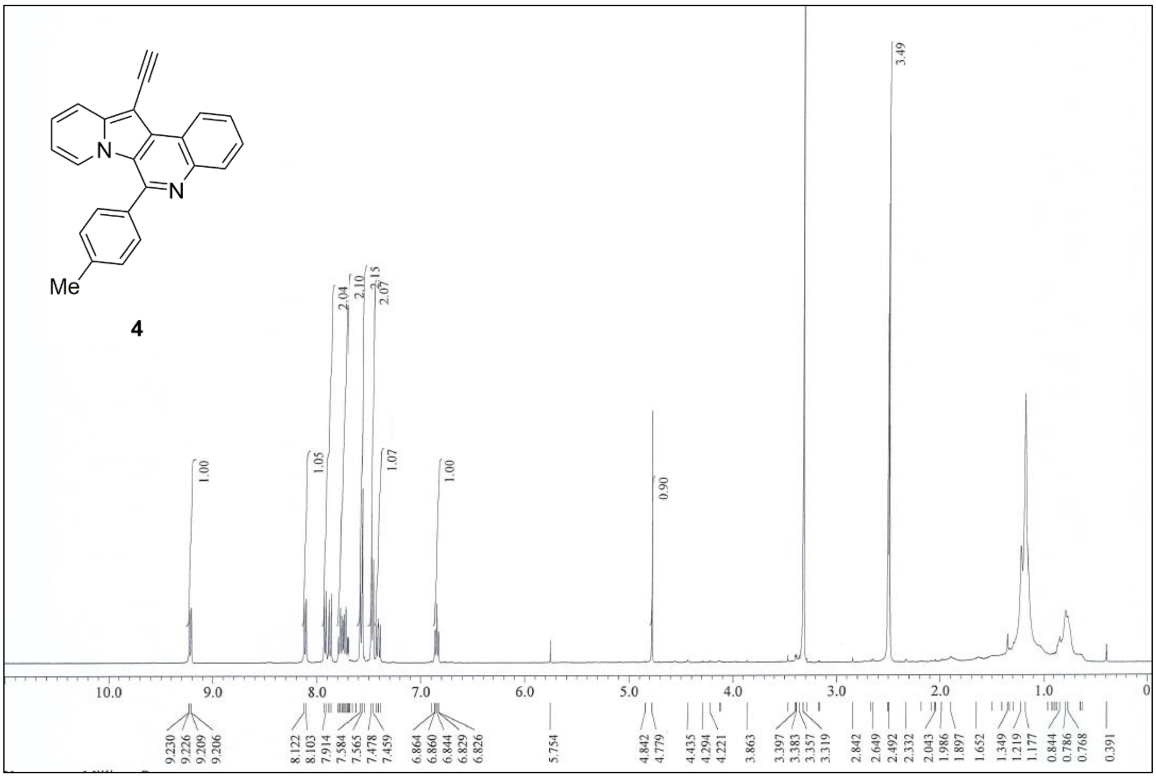

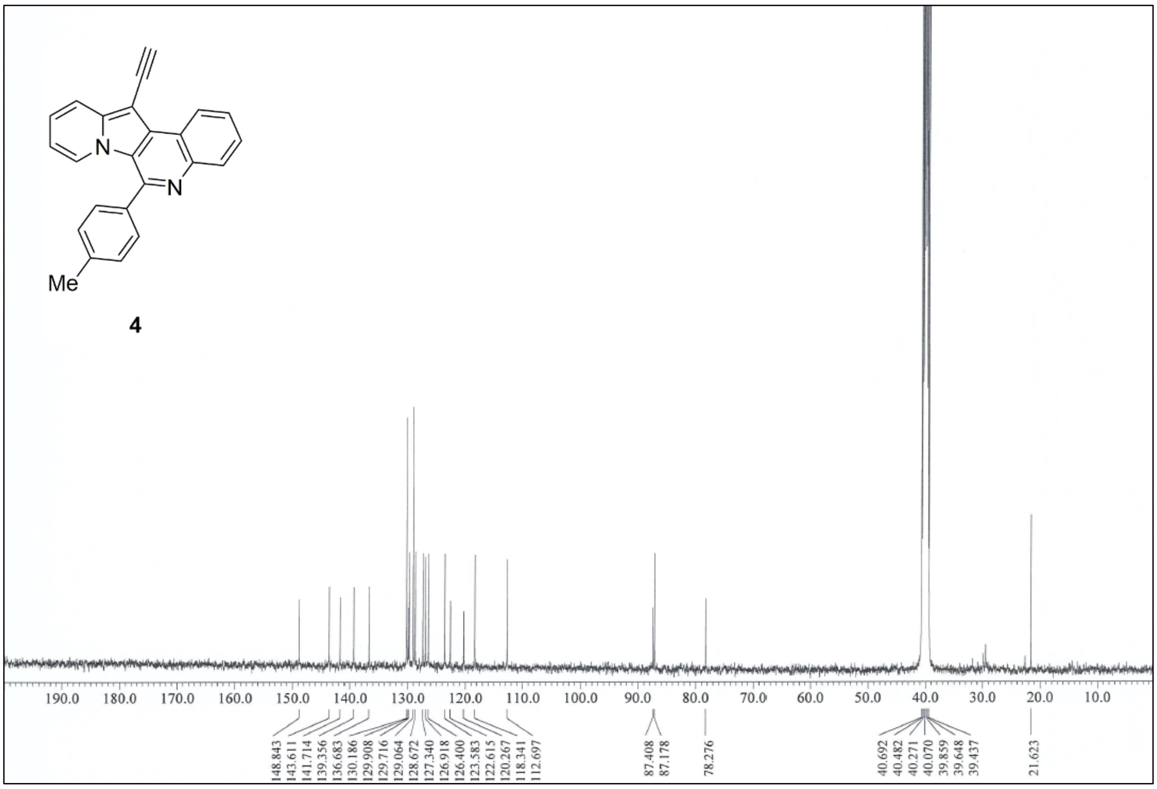

^{1}H NMR, ^{13}C NMR spectra for **5** (**PD3-B**)

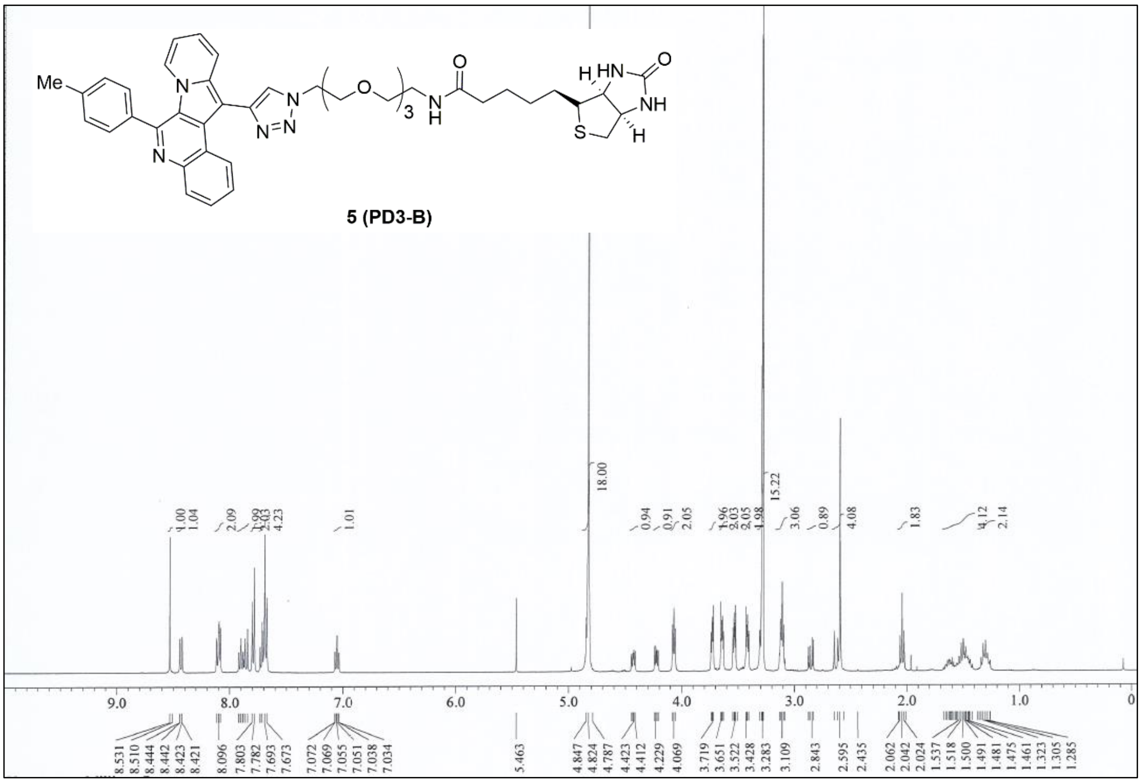

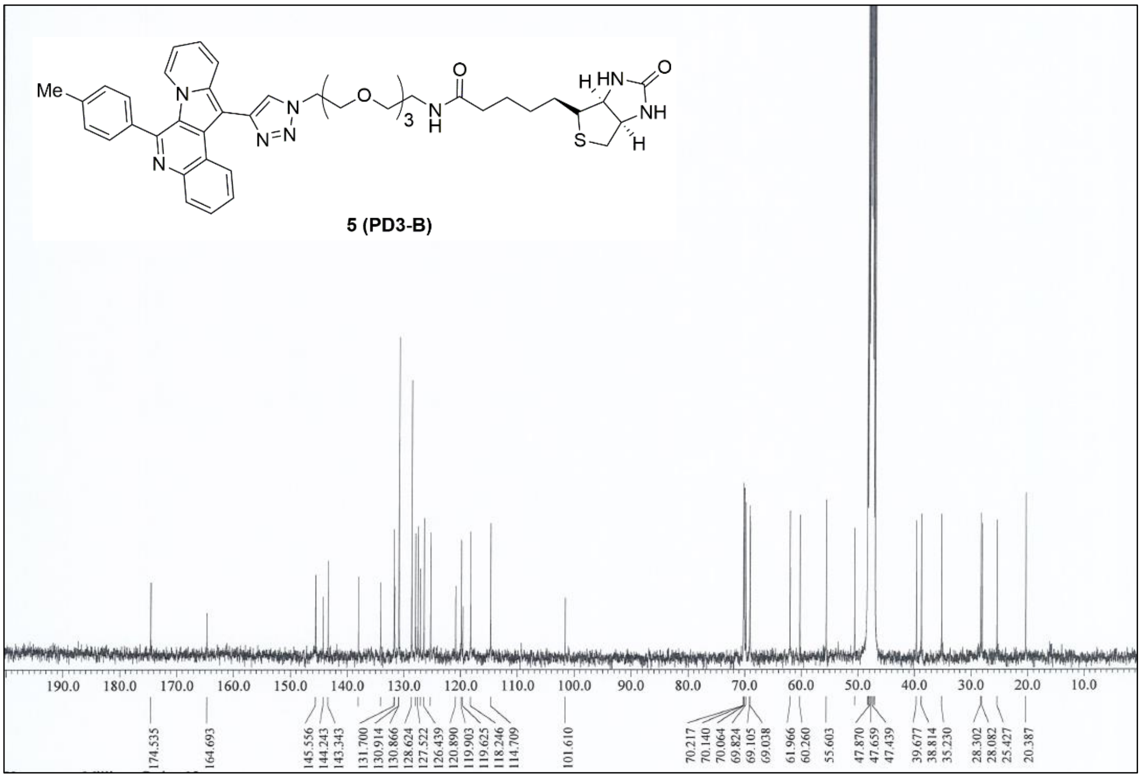

Supplement: Supplemental Material [file IENZ_A_2086865_SM1501.pdf]
